# Growing up as an Immunologist Among Molecular Geneticists

**DOI:** 10.1002/eji.70256

**Published:** 2026-08-17

**Authors:** Klaus Rajewsky

**Affiliations:** ^1^ Immune Regulation and Cancer Max‐Delbrück‐Center For Molecular Medicine in the Helmholtz Association (MDC) Berlin Germany

**Keywords:** biology, genetics, immunology, molecular biology, molecular genetics

## Abstract

When I started my independent career as a young medical doctor at the Institute for Genetics in Cologne in 1964, among a faculty of enthusiastic young scientists devoted to the then new field of molecular genetics, my hope to address the basic mysteries of immunology at a similar level was entirely speculative. But the situation changed dramatically over the next three decades, during which we witnessed, and contributed to, the incorporation of basic methods of bacterial and bacteriophage genetics into immunological research, including mutational analysis, ultimately developed into conditional targeted mutagenesis in the mouse. This was accompanied by revolutionary advances in cell separation, the advent of monoclonal antibodies, as well as nucleic acid amplification and sequencing technologies. The present article describes some of the activities of my group in Cologne over these times, focusing on how things got underway in this unique historical context, and along the lines of a lecture given in acceptance of the inaugural EFIS Lifetime Achievement Award in Vienna, 2025.

## Graphical abstract

The article describes how the author's immunological work and group developed among the molecular geneticists at the Institute for Genetics in Cologne, during the times of the foundation of molecular biology and European unification.

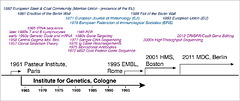

My first contact with immunology was at the Institut Pasteur in Paris, where I arrived as a young postdoc in 1962, in the laboratory of Pierre Grabar, and began to immunize rabbits with lactic dehydrogenase (LDH) isoenzymes, whose biochemical characterization had been the subject of my MD thesis at Frankfurt University in Germany. These were early times after the Second World War, with all the excitement of international connections opening up for young Germans of my generation. Raising and characterizing antibodies in rabbits exposed me to immunological concepts like self‐non‐self‐discrimination, immunological tolerance, learning and memory, which I soon found more exciting than isoenzyme biochemistry. In addition, luck had it that close to our lab was that of Francois Jacob and Jacques Monod, who at that time were involved in groundbreaking work on gene regulation in bacteria, earning them and Andre Lwoff a Nobel Prize a few years later. Their lab was populated by ambitious and lively students and postdocs, and often featured several in‐lab seminars per week, where I would occasionally sneak in and listen to their inspired and heated discussions. Without much understanding, I was deeply attracted by their world of molecular mechanisms and mutational approaches, their enthusiasm and high spirits.

Having returned from Paris to Frankfurt in 1964 to do some remaining clinical work in the frame of my medical studies, I had the good luck to be taken by a friend from Oxford I had met at Pasteur, Spedding Micklem, to a conference in Prague, then one of the world centers of immunology. This was again an eye‐opening experience, with not only Sir MacFarlane Burnet and many other leading immunologists being present, but also Niels Jerne, a later mentor of mine. Returning from Prague to my clinical work, I was invited to speak at a meeting of the German Biochemical Society, and after my talk Ulf Henning offered me a position at the newly founded Institute for Genetics at the University of Cologne.

This offer was special. A founding director of the new Genetics Institute was Max Delbrück, on visit from Caltech. Max had emigrated from Berlin to the USA in the mid‐thirties and became the founder of the famous phage group in California. His idea was to establish at the University of Cologne a new US‐style scientific center in post‐war Germany, with flat hierarchies, a cooperative spirit, and a focus on the world of bacterial and phage genetics. The new place turned out to be a magnet for young, ambitious scientists, many of them, including Ulf Henning, returning from the US. Its famous annual phage course became a must for biochemistry professors across the country, and the annual Spring Meetings of Molecular Biology that grew out of these courses became European events with many hundreds of participants. For me, Ulf Henning's offer was irresistible, as it gave me the opportunity to follow my own research interests in an internationally well‐connected environment and surrounded by molecular geneticists like the ones I had met in Paris, with the hope of studying immune reactions mechanistically, at the level of molecular biology. I abruptly ended my clinical work in Frankfurt and joined the Institute for Genetics in Cologne, still before the end of 1964.

In reality, the situation was a little scary, given that besides my own position (that of a substitute research assistant), a small lab and the position of a technician, I had 12 cages for rabbits at my disposal, no additional budget, and almost no immunology colleagues at the institute. In this situation, having used rabbit antibodies for the characterization of LDH isoenzymes at the Pasteur Institute, I turned my attention to the underlying immune response in these animals, besides writing grants to the Deutsche Forschungsgemeinschaft. The LDH isoenzymes had been shown to be tetrameric proteins consisting of two serologically distinct subunits, A and B, in all possible combinations. Immunizing rabbits with A2B2 tetramers from pig resulted in high and equal titers of anti‐A and anti‐B antibodies in the blood of the immunized animals. Analyzing these antibodies by serum immunoelectrophoresis combined with an LDH‐specific staining reaction as I had learned to do in Paris, I discovered that the antibodies raised against pig LDH cross‐reacted, albeit at low affinity, with the rabbit's own isoenzymes, which disappeared from their typical electrophoretic position in the serum of the immunized animals. How did this fit into the concept of immunological tolerance? In analogy to the classical experiments of Brent and Medawar on acquired tolerance in 1953, I soon found myself injecting newborn rabbits with a single dose of pig LDH isoenzymes, letting them grow up, and testing whether they would respond to LDH immunization as adults—and to my initial total disbelief, their response was indeed strongly reduced! But there were additional interesting twists to the experiment: Only the A4 tetramer from pig induced tolerance, while the B4 enzyme was inactive in that respect; and paradoxically, tolerance to the A4 tetramer extended equally to both the anti‐A and anti‐B response upon immunization of the adult animals with the A2B2 tetramer [1]! I began to think of the A2B2 LDH heterodimer as a hapten‐carrier complex, in which the B subunit behaved as a hapten, carried along by the A subunits. Tolerance to the A2B2 tetramer would be induced at the level of carrier (subunit A) recognition and would not extend to the cells specifically recognizing and producing antibodies against subunit B. Discussing this conceptual problem with Niels Jerne, who had moved to my hometown Frankfurt in 1966 and become my immunology mentor, the decision was made to switch experiments to classical hapten‐carrier conjugates, where one could experimentally combine a given hapten with distinct carriers. Experiments along these lines quickly confirmed that the antibody response to hapten‐carrier conjugates indeed required specific immunological recognition of both hapten and (separate) carrier determinants. In 1969 we published a paper in the Journal of Experimental Medicine, in which we suggested that the induction of an antibody response is based on the interaction of two antigen‐bridged cells [2], a first model of cell interactions in the immune system. Similar conclusions were reached by Avrion Mitchison [3], whom I had met at a Cold Spring Harbor Meeting in 1967 and who used a far more elegant cell‐transfer system between inbred mice in his studies, compared with my rabbit immunizations. Indeed, in 1969 I spent time as an EMBO Senior Fellow in Av's laboratory at Mill Hill, London, studying cell cooperation in the induction of antibodies with him through cell transfer in mice [4]; and being again exposed to an environment of exceptional scientists from all over the world, with lasting friendships developing. Upon my return from London, we substituted our rabbit colony with an ever‐growing colony of inbred mouse strains. By that time, work in Av's lab had provided evidence that hapten‐carrier cooperation reflected the cooperation of T and B cells [5], as we all had speculated—although the concept of the antigen bridge would later be replaced by antigen processing and peptide‐MHC recognition by the T cell antigen receptor.

At that time my little group in Cologne was getting noticed in the field and began to expand into several new directions, profiting from its unique integration into an environment of molecular genetics (with links to the University Hospital and the Physics Department) and the dramatic rise in the availability of molecular and cellular techniques for immunological research over the coming years. We were also given more space in the institute and began to recruit independent junior groups. Figure 1 highlights some of these developments, as well as our own activities, in the context of the general process of European unification which we were experiencing in those times. Relating to the latter was the foundation of the European Journal of Immunology in 1971, in which I myself participated, and of EFIS in 1975. With respect to our research interests those were revolutionary times: Immunoglobulin gene rearrangements explained antibody diversity, RNA and DNA sequencing and, ultimately, gene amplification by PCR as well as gene targeting in embryonic stem cells became available, monoclonal antibodies could be generated through hybridoma production, and fluorescence‐activated cell sorting (FACS), dramatically improved through the use of monoclonal antibodies, allowed the isolation and analysis of cells of the immune system at a new level.

**FIGURE 1:**
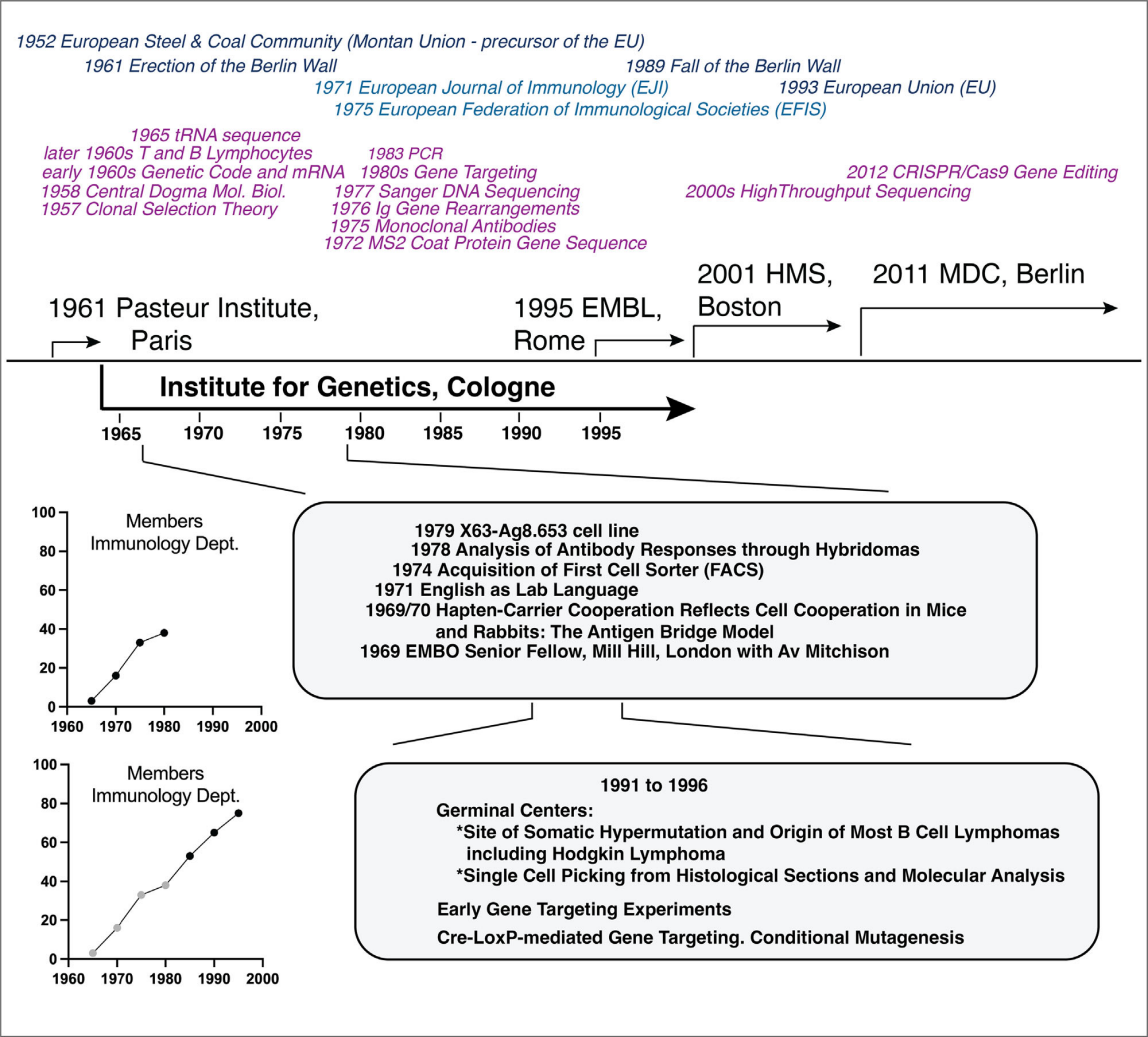
Key events and research activities of the author's group at the Institute for Genetics, Cologne, in their historical context. The top panel depicts steps toward European unification after World War II (blue) and major advances in the field of biological sciences over the same time period (magenta). The lower panels (black) highlight specific initial developments, from 1964 to the late 1970s (upper part), and work of the group from the 1990s (lower part). The diagrams on the left show the growth of the immunology department at the institute.

We were favorably placed to integrate these new opportunities into our thinking and research activities early on. Thus, through my connection with Len Herzenberg, whom I had met and befriended at a meeting in Hungary, we were able to buy, in 1974, what I think was the fourth FACS machine produced by Becton–Dickinson, and got it to work with the help of two students, Andreas Radbruch and Bernhard Liesegang. We also combined early on FACS with fluorescence‐labeled monoclonal antibodies specific for cell surface antigens, including monoclonal antibodies against major histocompatibility antigens generated in our own lab [6]. (In the latter context, we also contributed to the hybridoma technology as such, in that John Kearney, on sabbatical from Alabama, generated the X63‐Ag8.653 cell line [7], which had lost immunoglobulin (Ig) light chain expression and was subsequently used for hybridoma production all over the world, including the German Democratic Republic). With FACS and monoclonal antibodies at hand, we soon began to study the antibody response at a molecular level, along the lines of the work of the molecular geneticists in the institute: Andreas Radbruch addressed Ig class switch recombination by isolating class switch variants from a myeloma cell line [8], to later show that it was directed to the same switch regions on both the productive and the nonproductive Ig heavy chain allele in activated B cells [9]. Michael Reth dissected the antibody response to 3‐hydroxy‐4‐nitro‐phenylacetyl (NP) at the level of monoclonal antibodies [10], and in collaboration with Al Bothwell and David Baltimore found germ‐line variable (V) region sequences in primary and somatic mutation in secondary response antibodies [11]. Somatic mutation contributed to antibody affinity maturation, with later work showing that a single mutation, W33L, in the V region of the Ig heavy chain dominantly expressed in primary anti‐NP antibodies mediates a 10‐fold increase of affinity [12]. This mutation serves as a hallmark of antibody affinity maturation to this day.

The advent of gene amplification by the polymerase chain reaction (PCR) and of gene targeting in embryonic stem cells in the early 80s led to additional major new directions of our research activities and further growth of the immunological community at the institute (Figure 1). Using PCR, we could study somatic hypermutation of Ig V region genes at the level of small groups of, and ultimately single, cells; and gene targeting in embryonic stem cells of the mouse would potentially allow us to directly apply mutational analysis to the study of the immune response—my initial motivation to join the molecular geneticists in the institute in Cologne. Thus, spirits were high in the lab in those years, and we had a substantial influx of postdocs from many countries, particularly from Japan. The latter followed a tradition since the middle of the 70s, owing to my friendship with Tomio Tada, eminent scientist, writer, and artist. It had helped that I had early on, in 1971, introduced English as the common lab language, quite unusual in Germany at the time.

As a result of our new efforts, by the early 90s my own group and that of Claudia Berek, whom we had recruited to the institute, had independently established that somatic hypermutation of antibody V region genes took place in germinal centers (GCs). The Berek group had used flow‐cytometric isolation of GC cells for this purpose [13]; in my own group, collaborating with Joshy Jacob and Garnett Kelsoe from Duke, we scratched cells from histological sections of GCs and analyzed their genomic V gene rearrangements through PCR [14]. This was followed by the amplification of such rearrangements from single cells picked from histological sections, pioneered in the lab by Ralf Küppers, helped by the pathologist Martin‐Leo Hansmann, and leading to a first “spatial” single cell analysis in human GCs, and, in separate subsequent studies, the identification of Hodgkin lymphoma cells as transformed GC B cells [15, 16]. The latter work was embedded into a comprehensive analysis of human B cell lymphomas, most of which emerged as originating from the GC reaction based on their somatically mutated rearranged V region genes [17].

In parallel to these efforts, targeted mutagenesis in mouse embryonic stem (ES) cells became a top priority in the lab. As soon as we heard of these cells and their ability to form blood islands in vitro, in a seminar Rolf Kemler gave at the institute in 1986, we sent a postdoc, Nobuaki Yoshida, to Kemler's lab in Tübingen to learn how to handle those cells, and were soon able to generate chimeric mice producing ES cell‐derived lymphocytes through ES cell injection into blastocysts. Thus, when Thomas and Capecchi published their famous gene targeting paper in 1987 [18], we were ready to go, and immediately set up a small group of interested lab members, with weekly meetings in our “ES cell club”, to try to introduce targeted mutations into those cells, with subsequent transmission into the mouse germ line, or at least their analysis in chimeric mice through blastocyst complementation. It was again in the early 90s that the first results from these efforts became available: The µMT mouse of Daisuke Kitamura, showing that membrane expression of the Ig heavy chain of class µ, and thus of a µ‐chain‐associated receptor complex, is essential for B cell development [19] as well as heavy chain allelic exclusion [20]; and the knockout of the Ig‐δ heavy chain gene by Jürgen Roes, initially analyzed in the chimera system [21].

The generation and analysis of gene knockouts became a major activity in immunological research in those years. Our own efforts in this direction soon went beyond the analysis of B cell responses, addressing the role of cytokines in immune regulation (with IL10 deficiency causing enterocolitis [22]) and other matters. I will not go into this work here, nor describe other activities in the lab, such as the analysis of life spans, proliferation and selection of germ line versus somatically mutated antibody V regions in the development of B lymphocyte subsets of mouse and human, using BrdU incorporation as an additional tool (see [23, 24, 25, 26], and the identification of long‐lived plasma cells by the Radbruch group [27]), or our extensive, and in the end inconclusive, studies of idiotypic networks. Instead, I will end with our efforts to refine the gene targeting approach, to allow targeted mutagenesis in specific biological contexts. Along these lines, Shinsuke Taki inserted a (well‐known) Ig heavy chain V region rearrangement into its physiological position in the Ig heavy chain locus, to create a first second‐generation Ig transgenic mouse line [28]; and Yong‐Rui Zou exchanged the mouse by the human Ig κ light and Ig γ1 heavy chain constant region genes, with the resulting mice producing “humanized” IgG1 antibodies [29, 30]. Most consequential, however, were experiments in which we used the bacteriophage‐derived Cre‐loxP recombination system, earlier shown to be able to mediate recombination in mammalian cells [31], as a tool in gene targeting experiments in mice. Those experiments, initiated in the laboratory by Hua Gu in 1991, allowed “clean” targeted mutagenesis through Cre‐mediated elimination of selection marker genes used for mutant selection [32], and “conditional” mutagenesis, that is, the introduction of targeted mutations into specific cell types [33] and/or at specific times in development [34]. The study by Gu et al. [33] was a collaborative effort with Jamey Marth in Vancouver, who contributed a T cell‐specific cre transgenic mouse line which he had generated in experiments using Cre‐mediated recombination for transgene modification, in parallel with Heiner Westphal's group at NIH [35, 36]. It was the first demonstration that the problem of lethality of the knock‐out of a genomic gene in the germ line could be overcome by conditional gene inactivation. Cre‐mediated recombination also added gene inversion to the repertoire of targeted mutagenesis, first used in the lab in combination with Cre‐mediated deletion in the Ig heavy chain locus, to show that the persistence of memory B cells is independent of persisting immunizing antigen [37].

Conditional gene targeting in mice was soon used by many research groups worldwide, with the “Cre Zoo”, as we called it, growing to hundreds of cre transgenic strains. It also became a major research tool in our own work in Cologne and later on, starting with the demonstration that antigen receptor expression is vital for B cell maintenance [38]; but this is beyond the scope of the present article.

To conclude, starting with uncertain perspectives in 1964, immunological work had become a central activity at the Cologne Genetics Institute 30 years later. This became possible because new techniques developed over those years allowed us to study the immune system at the level of molecular cell biology and genetics. I am infinitely grateful to my many collaborators as well as the members of associated groups at the institute, who made this happen. Only a few of their names could be mentioned in this short article, not to speak of all our dedicated technical and administrative staff. A more detailed account of our activities at the Institute for Genetics can be found in reference [39]. As shown in Figure 1, in 2001, with obligatory retirement at the university looming, I moved my group from Cologne to Harvard Medical School in Boston, and 10 years later to the Max‐Delbrück‐Center in Berlin, where this article is written. My sincere thanks also go to EFIS for honoring me with its inaugural Lifetime Achievement Award, and to the many funding agencies which have supported our work over the years.

## Conflicts of Interest

The author declares no conflicts of interest.

## Data Availability

Data sharing not applicable to this article as no datasets were generated or analyzed during this study.
